# Regulation of cell death in cancer—possible implications for immunotherapy

**DOI:** 10.3389/fonc.2013.00029

**Published:** 2013-02-21

**Authors:** Simone Fulda

**Affiliations:** Institute for Experimental Cancer Research in Pediatrics, Goethe-University FrankfurtFrankfurt, Germany

**Keywords:** cell death, signal transduction, immunotherapy, childhood cancer

## Abstract

Since most anticancer therapies including immunotherapy trigger programmed cell death in cancer cells, defective cell death programs can lead to treatment resistance and tumor immune escape. Therefore, evasion of programmed cell death may provide one possible explanation as to why cancer immunotherapy has so far only shown modest clinical benefits for children with cancer. A better understanding of the molecular mechanisms that regulate sensitivity and resistance to programmed cell death is expected to open new perspectives for the development of novel experimental treatment strategies to enhance the efficacy of cancer immunotherapy in the future.

## Introduction

Programmed cell death is an intrinsic cellular program that is present in every cell of the body and involved in the regulation of various physiological and pathophysiological processes (Lockshin and Zakeri, 2007). Also, programmed cell death is evolutionary highly conserved underlining its critical role in the regulation of tissue homeostasis, a subtle balance in the maintenance of cell death and proliferation signals (Lockshin and Zakeri, 2007). In addition, the antitumor activity of most anticancer therapies, including immunotherapy, critically relies on the induction of programmed cell death in cancer cells. However, cell death programs are typically blocked in human cancers, since the evasion of cell death provides a survival advantage to the tumor (Fulda, 2009b). This implies that the efficacy of antitumor therapies, e.g., immunotherapy, is impaired by the inactivation of cell death pathways in tumor cells. Therefore, one strategy to enhance the efficacy of cancer immunotherapy resides in the reactivation of cell death pathways in tumor cells. By lowering the threshold to trigger cell death in cancer cells, it is anticipated that immunotherapies will be more effective in killing their target cells. This concept implies that a better understanding of the molecular mechanisms that regulate cell death programs in cancer cells will likely yield novel targets for therapeutic intervention that can be used to augment immunotherapy-based anticancer strategies. This approach may open new perspectives to improve the antitumor activity of immunotherapies.

## Programmed cell death

The first description of programmed cell death dates back to the mid-1960s (Kerr, 1965; Lockshin and Williams, 1965). Since then several forms of programmed cell death have been identified, including apoptosis, necroptosis, or autophagic cell death (Galluzzi et al., 2012). Apoptosis represents one of the best characterized modes of cell death that is highly conserved throughout evolution and involved in the regulation of various physiological conditions. In addition, there is a huge body of evidence demonstrating that deregulation of apoptosis contributes to various human diseases (Lockshin and Zakeri, 2007). For example, too little apoptosis can promote tumor formation and progression and also plays a critical role in conferring treatment resistance (Fulda, 2009b). Necroptosis has recently been identified as a regulated, caspase-independent mode of cell death (Vandenabeele et al., 2010). In contrast to necrosis that represents an accidental form of cell death, necroptosis is classified as a programmed form of necrosis that is often engaged under conditions of insufficient caspase activation (Vandenabeele et al., 2010). Recently, necroptosis has been reported as an alternative cell death program that is triggered in apoptosis-resistant acute leukemia cells that lack FADD or caspase-8 (Laukens et al., 2011), indicating that necroptosis may provide a new approach to overcome apoptosis resistance. Autophagic cell death is characterized by the dependence on autophagy genes for its execution along with typical morphological features such as cytoplasmic vacuolization (Galluzzi et al., 2012). The current review focuses on apoptosis, since its implication in the regulation of immunotherapy-induced cell death has most extensively been studied.

## Death receptors

Death receptors are part of the superfamily of tumor necrosis factor (TNF) receptors, a large family of transmembrane receptors that exhibit a broad spectrum of biological activities, including the control of programmed cell death and immune functions (Ashkenazi, 2008). The unifying structural feature of the death receptor family resides in a cytoplasmic domain, i.e., the “death domain” (Ashkenazi, 2008). This protein stretch of about 80 amino acids mediates protein–protein interactions and is critically required for the transduction of the lethal signal from the outside to the interior of the cell (Ashkenazi, 2008). As far as the induction of cell death is concerned, two death receptor systems have been best characterized, i.e., the CD95 (APO-1/Fas) system and the TNF-related apoptosis-inducing ligand (TRAIL) receptor system. Both receptor systems comprise transmembrane cell surface receptors that harbor the intracellular death domain and a cysteine-rich extracellular domain that serves for binding of cognate ligands (Ashkenazi, 2008). While one CD95 receptor is known, four distinct membrane-based TRAIL receptors (TRAIL-Rs) have been identified in the mammalian system (Ashkenazi, 2008). Two of these TRAIL-Rs signal to cell death, i.e., TRAIL-R1 and TRAIL-R2, whereas TRAIL-R3 and TRAIL-R4 represent antagonistic receptors that do not signal to cell death, although they are able to bind TRAIL as the corresponding ligand (Ashkenazi, 2008). This higher level of complexity in the TRAIL-R/ligand system has resulted in the generation of specific monoclonal antibodies that specifically target the agonistic TRAIL-Rs TRAIL-R1 and TRAIL-R2. The CD95 receptor/CD95 ligand system plays an important role in the regulation of immune function (Ehrenschwender and Wajant, 2009). For example, the CD95 system contributes to the control of the adaptive immune response by mediating activation-induced cell death (AICD) of T cells. This implies that the regulation of CD95 signaling may have an impact on tumor formation and progression. In addition to CD95/CD95 ligand, also TRAIL is expressed by various cells of the immune system, including natural killer (NK) cells, T cells, dendritic cells, and macrophages (Falschlehner et al., 2009). TRAIL has been shown to be involved in the regulation of immunoregulatory functions and immune surveillance of tumors and metastasis. Results derived from studies using TRAIL knockout mice have shown that TRAIL exerts a crucial role in tumor immune surveillance (Smyth et al., 2001, 2003; Takeda et al., 2001; Cretney et al., 2002; Finnberg et al., 2008; Grosse-Wilde et al., 2008). Of note, lack of TRAIL or its receptors was shown to be associated with increased susceptibility to tumor metastasis compared to wild-type animals (Cretney et al., 2002; Finnberg et al., 2008; Grosse-Wilde et al., 2008). Furthermore, TRAIL expression on NK cells was reported to restrain metastatic spread of tumor cells (Smyth et al., 2001; Takeda et al., 2001). In addition, the TRAIL system has been implicated in the regulation of carcinogenesis. To this end, it was shown that carcinogenesis-triggered cancer formation was increased in mice lacking the TRAIL-R or in the presence of antagonistic TRAIL-R antibodies (Takeda et al., 2001; Finnberg et al., 2008). These studies imply that the TRAIL-R/ligand system plays an important role in the regulation of tumor immune surveillance during both tumor formation and progression. Thus, resistance to TRAIL-induced apoptosis may favor tumor immune escape.

## Apoptosis signaling pathways

**Figure 1:**
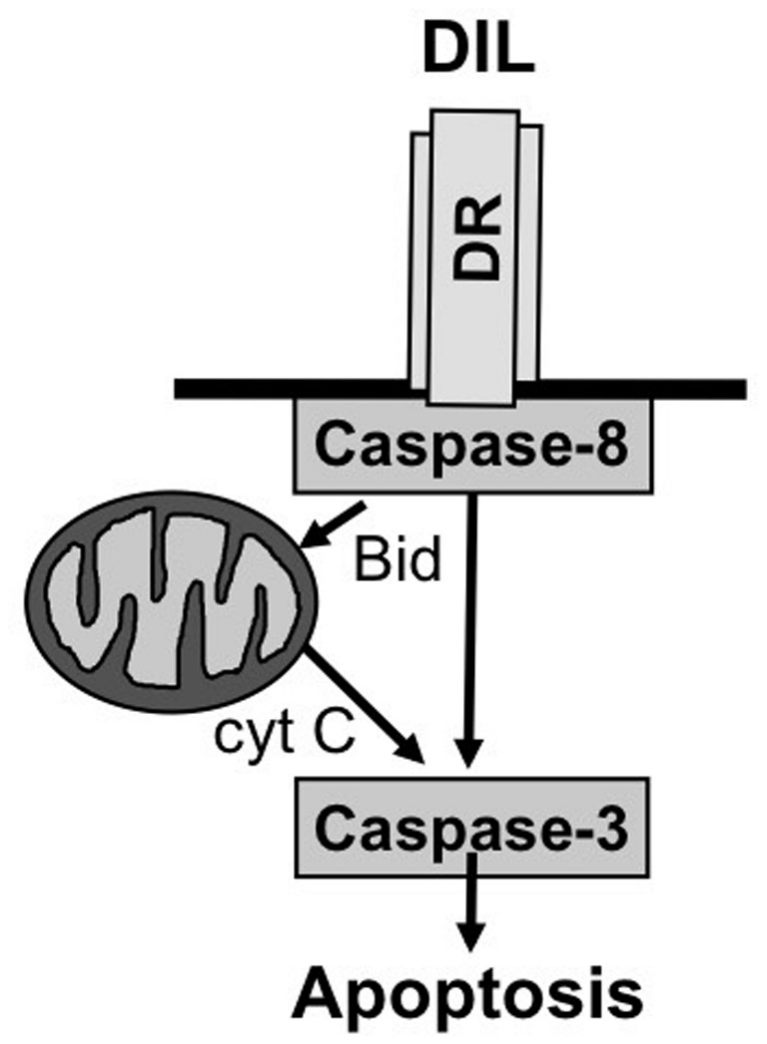
**Core apoptosis signaling pathways.** See text for more details.

Two principal signal transduction pathways leading to the induction of apoptosis have been delineated, i.e., the receptor (extrinsic) pathway and the mitochondrial (intrinsic) pathway of apoptosis (Fulda and Debatin, 2006b) (Figure 1). Engaging the apoptotic machinery via either of these two routes results eventually in the activation of caspases, a family of cysteine proteases that function as death effector molecules in various forms of apoptosis by proteolytic cleavage of multiple cytoplasmic or nuclear substrates (Degterev et al., 2003). This proteolytic breakdown of intracellular material including cytoskeletal proteins and nuclear DNA eventually results in the organized breakdown of the cell, typically without any spillage of the intracellular content into the environment.

As far as the death receptor (extrinsic) pathway of apoptosis is concerned, this cascade is typically engaged by the binding of death receptor ligands to their cognate death receptor on the cell surface (Ashkenazi, 2008). This leads to the oligomerization of death receptors into aggregates and subsequent recruitment of adapter and signaling molecules to activated death receptors to form the so-called death-inducing signaling complex (DISC). The assembly of this multi-protein complex facilitates the activation of the initiator caspase-8 via induced proximity. Once activated, caspase-8 can trigger the activation of effector caspases and apoptosis either directly or indirectly. In a direct manner, caspase-8 cleaves caspase-3 as one of the effector caspases which in turn results in proteolytic processing of substrates that mediate the dismantling of the cell and nuclear fragmentation. In an indirect manner, caspase-8 can engage the mitochondrial pathway of apoptosis by proteolytic cleavage of Bid into its activated form, i.e., truncated Bid (tBid) (Adams and Cory, 2007). Bid is one of the proapoptotic proteins of the Bcl-2 family of proteins that is characterized by only one BH3 domain (Adams and Cory, 2007). Once cleaved, tBid translocates from an intracellular cytoplasmic pool to mitochondrial membranes where it promotes the permeabilization of the outer mitochondrial membrane. Thereby, tBid engages the mitochondrial pathway of apoptosis which cumulates in the release of mitochondrial intermembrane proteins into the cytosol, including cytochrome c and second mitochondrial activator of caspases (Smac) (Fulda et al., 2010). In the cytosol, cytochrome c assembles together with apoptotic protease activating factor (Apaf)-1 and caspase-9 in a multimeric protein complex called the apoptosome that facilitates activation of caspase-9 and subsequently caspase-3. Smac promotes apoptosis by binding and neutralizing Inhibitor of Apoptosis (IAP) proteins, a family of proteins that negatively regulate apoptosis (Fulda and Vucic, 2012).

It is important to know that cell death signaling pathways to apoptosis are tightly controlled in normal and malignant cells, given the fact that accidental activation of cell death pathways might have a detrimental effect on cell survival (Fulda, 2009b). Thus, there are various proteins that positively or negatively regulate signal transduction to apoptosis at various stages of the signal transduction cascade. Importantly, these inbuilt regulatory mechanisms to control programmed cell death are typically dysregulated in human cancers in such a way that the ratio of pro- and antiapoptotic signals is tilted toward factors that block signal transduction to cell death (Fulda, 2009b).

## Defects in cell death pathways in human cancers

A hallmark of human cancers is their tendency to evade programmed cell death, since the ability to resist the induction of cell death provides a survival advantage to malignant cells (Hanahan and Weinberg, 2011). On theoretical grounds, resistance to programmed cell death can be caused by loss of expression or function of proapoptotic molecules and/or by aberrantly high expression levels of proteins that inhibit programmed cell death (Fulda, 2009b).

## Mechanisms of resistance to cell death

Death receptor-induced apoptosis may be blocked by downregulation of surface expression levels of death receptors, including CD95 and TRAIL-Rs (Friesen et al., 1997; Fulda et al., 1998). Also, mutational inactivation of death receptors can contribute to the resistance to death receptor-mediated apoptosis. For example, mutations of CD95 have been detected in both B cell and T cell acute lymphoblastic leukemia (ALL) (Beltinger et al., 1998a,b). In addition, the chromosomal region on chromosome 8p which harbors the genetic localization of both agonistic TRAIL-Rs is frequently inactivated in human cancers via loss of heterozygocity (LOH) (Emi et al., 1992; Wistuba et al., 1999). In addition to mutational inactivation of death receptors, epigenetic events can also contribute to silencing of death receptor expression levels. CD95 as well as TRAIL-Rs have been reported to be among the targets of epigenetic silencing via hypermethylation of CpG-island-rich regions of the promoters of CD95 or TRAIL-Rs (Van Noesel et al., 2002; Petak et al., 2003).

Besides death receptors, also the initiator caspase caspase-8 is often epigenetically inactivated in human cancers, which similarly confers resistance to receptor-mediated apoptosis (Teitz et al., 2000; Fulda et al., 2001; Hopkins-Donaldson et al., 2003). Furthermore, death receptor-mediated apoptosis can be impaired by a splice variant of caspase-8, i.e., caspase-8L. This caspase-8 variant is produced via alternative splicing and blocks death receptor-induced apoptosis in a dominant-negative manner (Mohr et al., 2005; Miller et al., 2006).

Death receptor-triggered programmed cell death can also be blocked via aberrant upregulation of antiapoptotic proteins. In principle, negative regulation of signal transduction along the death receptor pathway can be interrupted at distinct levels of the signaling cascade. For example, cellular FLICE-inhibitory protein (cFLIP) prevents death receptor signaling by interfering with caspase-8 activation at the level of the DISC (Micheau, 2003; Fulda, 2013). Furthermore, the balance between pro- and antiapoptotic proteins of the Bcl-2 family is typically disturbed in human cancers. Bcl-2 proteins comprise both proapoptotic as well as antiapoptotic family members (Adams and Cory, 2007). Overexpression of the antiapoptotic Bcl-2 proteins such as Bcl-2, Bcl-X_L_, and Mcl-1 frequently occurs in human malignancies, whereas the proapoptotic family members are downregulated or inactivated (Adams and Cory, 2007). For example, somatic mutations of the Bax gene have been reported in colon carcinoma or hematological malignancies (Rampino et al., 1997; Kitada et al., 2002).

IAP proteins represent another family of antiapoptotic proteins that negatively regulate signal transduction to programmed cell death (Fulda and Vucic, 2012). IAP proteins are expressed at high levels in various human cancers and have been correlated with resistance to cell death and poor prognosis (Fulda and Vucic, 2012). Among the IAP proteins, it is in particular X-linked inhibitor of apoptosis protein (XIAP) that blocks signaling to programmed cell death by binding to and inhibiting caspases such as caspase-3, -7, and -9 (Eckelman et al., 2006; Fulda and Vucic, 2012).

## Experimental approaches to restore cell death signaling pathways in human cancers

In light of the fact that cell death pathways are frequently disturbed in human cancers, which has been linked to treatment resistance including resistance to immunotherapies, there have been major efforts to develop experimental strategies to restore cell death signaling pathways in human cancers. In principle, this can be achieved by upregulation of expression levels of proapoptotic molecules and/or neutralization of antiapoptotic proteins that are aberrantly expressed.

Since death receptors such as CD95 and TRAIL-Rs are under the control of the tumor suppressor gene p53, one approach to upregulate surface expression of death receptors resides in the use of DNA-damaging agents that activate p53. To this end, concomitant administration of anticancer drugs or ionizing radiation together with death receptor ligands resulted in cooperative induction of cell death via increased surface expression of death receptors (Gliniak and Le, 1999; Chinnaiyan et al., 2000; Nagane et al., 2000).

Since caspase-8 represents a key component of the death receptor pathway which is frequently silenced in human cancers, restoration of caspase-8 expression provides an alternative approach to restore defective cell death programs (Fulda, 2009a). To this end, demethylating agents such as 5-axa-2-deoxycytidine have been demonstrated to cause demethylation of the regulatory region of caspase-8 and increased caspase-8 expression levels, which in turn resulted in restoration of sensitivity toward death receptor-mediated cell death (Hopkins-Donaldson et al., 2000; Teitz et al., 2000; Fulda et al., 2001). In addition, interferon-γ has been identified as a cytokine that is involved in the regulation of caspase-8 expression levels, as the caspase-8 promoter harbors interferon-γ activation sites (Fulda and Debatin, 2002; Casciano et al., 2004; Fulda and Debatin, 2006a; Hacker et al., 2009).

An alternative strategy to restore cell death signaling pathways is the therapeutic targeting of antiapoptotic Bcl-2 proteins. To this end, several small-molecule inhibitors directed against antiapoptotic Bcl-2 family proteins have been developed (Fulda et al., 2010). One of the most prominent examples is ABT-737 and its derivative, a small-molecule inhibitor that binds to Bcl-2, Bcl-X_L_, and Bcl-w (Oltersdorf et al., 2005). Besides small-molecule inhibitors of antiapoptotic Bcl-2 proteins, also BH3 peptides have been designed similarly to the structure of BH3-only domain proteins (Ni Chonghaile and Letai, 2008). Also, antisense oligonucleotides directed against Bcl-2 were shown to downregulate Bcl-2 mRNA expression and to enhance sensitivity to cell death in response to chemotherapeutic treatment (Tolcher et al., 2005).

Another strategy to enhance sensitivity to cell death resides in the therapeutic neutralization of IAP proteins (Fulda and Vucic, 2012). To this end, antisense strategies directed against XIAP have been developed which proved to enhance cell death induction either alone or in combination therapies (Lacasse et al., 2005; Lacasse, 2012). In addition, small-molecule IAP inhibitors such as Smac mimetics were shown to either directly trigger cell death or to sensitize cancer cells for death receptor-mediated apoptosis (Fulda et al., 2002; Fakler et al., 2009; Vogler et al., 2009; Abhari et al., 2012; Basit et al., 2012).

## Conclusions

Programmed cell death is an intrinsic cellular program that regulates various physiological processes and is typically disturbed in human cancers. Since the efficacy of current cancer therapies critically relies on the engagement of this cell intrinsic program, defects in programmed cell death form the basis for treatment resistance. This implies that defective cell death signaling pathways can dampen the efficacy of cancer immunotherapies. Therefore, further insights into the regulation of programmed cell death in cancer cells are expected to pave new avenues for the development of more effective treatment approaches based on the modulation of the immune systems in cancer patients. One example is the combination of cellular immunotherapy approaches together with molecular strategies. Thus, incorporation of the advances in cell death research in the concepts of cancer immunotherapies will likely boost this important field in the near future.

### Conflict of interest statement

The research was conducted in the absence of any commercial or financial relationships that could be construed as a potential conflict of interest.
